# Flexural Capacity Calculation Method for Pre-Damaged RC Beam–Column Joints Strengthened with High-Strength Steel Strand Mesh-Reinforced ECC

**DOI:** 10.3390/ma19184002

**Published:** 2026-09-20

**Authors:** Shuaijun Liu, Yaoxin Wei, Ziyuan Li, Jiahao Hu, Ke Li, Wei Li, Shasha Xu

**Affiliations:** 1 Department of Civil Engineering, Zhengzhou College of Finance and Economics, Zhengzhou 450044, China; liushuaijun8367@163.com; 2 Department of Civil Engineering, Zhengzhou University, Zhengzhou 450001, Chinairwinlike@163.com (K.L.); 3 SINO-SINA Building Materials Co., Ltd., Zhengzhou 452370, China

**Keywords:** RC beam–column joint, high steel strand mesh-reinforced ECC, flexural behavior, pre-existing damage

## Abstract

Pre-existing damage in reinforced concrete (RC) beam–column joints may induce strain incompatibility between the original member and subsequently applied strengthening layers, limiting the applicability of conventional flexural-capacity models. This study experimentally and analytically investigates pre-damaged RC beam–column joints strengthened with high-strength steel strand wire mesh-reinforced engineered cementitious composite (HSSWM–ECC). Eight specimens were tested to evaluate the effects of pre-damage level, steel strand spacing, and axial compression ratio on beam-end flexural behavior. HSSWM–ECC effectively suppressed crack localization and concrete spalling, and all strengthened specimens ultimately exhibited beam-end flexural failure while the columns and joint cores remained largely intact. Compared with the unstrengthened specimen, the peak load increased by 39.24–63.88%. Reducing the strand spacing from 70 to 30 mm increased the peak load by 8.86%, whereas the higher pre-damage level caused an 11.28% reduction relative to the undamaged strengthened specimen. Within the investigated axial compression ratio range of 0.2–0.4, the difference in peak load remained below 3.64%, whereas increasing axial compression was associated with more pronounced post-peak strength degradation. Based on sectional force equilibrium and the plane-section assumption, an analytical model was developed by incorporating damage-induced strain lag and the contribution of the wrap-around side strengthening layers. The predicted capacities of the seven strengthened specimens agreed well with the experimental results, with deviations within 7%. The proposed model provides a rational approach for evaluating the beam-end flexural capacity of pre-damaged RC beam–column joints strengthened with HSSWM–ECC within the investigated parameter range and flexure-dominated failure mode.

## 1. Introduction

Existing reinforced concrete (RC) frame structures often exhibit varying degrees of pre-existing damage and deterioration in load-carrying capacity owing to outdated design provisions, long-term environmental exposure, and accidental loading [1]. For structures that retain a certain level of serviceability, repair and strengthening to restore their structural capacity generally offer greater economic and sustainability benefits than demolition and reconstruction [2,3,4]. Beam–column joints are critical regions in RC frames, where internal forces are transferred, and deformation compatibility between structural members is maintained; their mechanical response therefore has a direct influence on the overall structural safety. In practice, joints requiring strengthening commonly exhibit pre-existing cracking, stiffness degradation, and residual deformation. Such damage not only reduces the residual load-carrying capacity of the beam-end section but may also induce strain incompatibility between the existing RC section and the subsequently added strengthening layer under reloading. Accordingly, reliable evaluation of the flexural capacity of strengthened beam ends in the presence of pre-existing damage is a critical issue in the rehabilitation design of existing RC frame structures.

Damaged RC beam–column joints are commonly rehabilitated using concrete jacketing, steel-based strengthening, or externally bonded fiber-reinforced polymer (FRP) systems [5,6,7]. Among cementitious retrofit techniques, thin RC or shotcrete jackets incorporating distributed steel reinforcement are particularly attractive because they can restore local resistance and improve crack control while limiting the increase in member dimensions. Karayannis et al. [8] investigated damaged exterior RC beam–column joints locally retrofitted with thin RC jackets reinforced by small-diameter steel bars and demonstrated substantial recovery of strength and deformation capacity. Tsonos [9] further showed that reinforced shotcrete jacketing can effectively improve the response of deficient RC columns and beam–column joints. These studies demonstrate the effectiveness of combining a relatively thin cementitious layer with distributed steel reinforcement; however, the performance of such systems may still be influenced by interface integrity, deformation compatibility, and localized damage development. Engineered cementitious composites (ECCs), characterized by tensile strain-hardening and multiple-cracking behavior, have therefore attracted increasing attention for the repair and strengthening of existing RC structures [10]. Chidambaram and Agarwal [11] reported improved load-carrying capacity, stiffness, and energy dissipation in beam–column joints incorporating fiber-reinforced cementitious composites, while Suryanto et al. [12] showed that the use of ECC in exterior joints mitigated damage concentration and promoted a more distributed cracking response. Hung et al. [13] further demonstrated the potential of ECC-based jacketing for joint retrofit through comparison with conventional concrete- and FRP-based strengthening schemes. At the member level, ECC strengthening has also been shown to enhance the flexural response and deformation compatibility of RC beams [14,15]. Collectively, these studies highlight the advantages of ECC in crack control and damage redistribution, although the additional tensile resistance provided by ECC alone may remain limited when substantial enhancement of beam-end flexural capacity is required.

To provide a more effective tensile load-transfer path, high-strength steel strand wire mesh (HSSWM) can be embedded within ECC to form an HSSWM–ECC strengthening system. The steel strand mesh primarily provides additional tensile resistance, whereas the ECC matrix promotes distributed cracking, protects the embedded strands, and facilitates stress transfer between the strengthening layer and the existing concrete. Zhang et al. [16] investigated the interfacial bond behavior between HSSWM–ECC and concrete and established an analytical description of the corresponding bond response. At the structural-member level, Li et al. [17] examined the flexural behavior of RC beams strengthened with steel wire rope mesh-reinforced ECC and developed approaches for evaluating their flexural response, while Yang et al. [18] extended this concept to damaged RC beams strengthened with steel strand meshes and ECC. The influence of prior loading has also been considered in related strengthened-beam studies. Wei et al. [19] showed that secondary loading can introduce strain lag between the original member and the subsequently applied strengthening system, whereas Zhao et al. [20] demonstrated that residual strain after unloading can affect the subsequent flexural and shear response of repaired RC beams. These studies establish an important basis for understanding HSSWM–ECC strengthening at the interface and member levels. Nevertheless, most existing analytical approaches have been developed for isolated beams and generally consider the tensile strengthening layer under simplified deformation-compatibility assumptions. The combined influence of pre-existing damage and the side-layer contribution associated with wrap-around strengthening of beam–column joints has not yet been adequately addressed.

For pre-damaged RC beam–column joints, these two effects are mechanically coupled: pre-existing damage and unloading may delay mobilization of the subsequently applied HSSWM–ECC layer, while the wrap-around configuration introduces side strengthening layers that also participate in sectional force transfer. The key analytical challenge is therefore to represent both effects within a unified beam-end flexural-capacity formulation. To address this issue, eight RC beam–column joint specimens were tested monotonically with pre-damage level, steel strand spacing, and column axial compression ratio as the principal variables. Based on the experimentally identified beam-end flexural mechanism, a sectional capacity model was developed by incorporating a damage-related modification of the HSSWM–ECC contribution together with an equivalent representation of the side strengthening layers. The proposed formulation extends existing member-level approaches to pre-damaged HSSWM–ECC-strengthened beam–column joints and is assessed against the measured capacities within the investigated parameter range and beam-end flexural failure mode.

## 2. Test Program

### 2.1. Specimen Design

According to the Code for Design of Concrete Structures [21], a total of eight reinforced concrete beam–column joint specimens were designed and fabricated, including one unstrengthened control specimen and seven specimens strengthened with high-strength steel strand mesh-reinforced ECC. Three key variables were investigated in the experimental program, namely the pre-damage level, steel strand mesh spacing, and axial compression ratio. The specimen configuration and corresponding design parameters are summarized in Table 1.

It should be noted that one specimen was tested for each parameter combination in the present experimental program. Therefore, specimen-to-specimen variability cannot be quantified statistically, and the comparisons presented herein are intended to identify the response trends within the investigated parameter ranges rather than to establish statistically generalized parameter effects.

The axial compression ratio, *n*, was defined as *n* = *N*/(*f_c_A_c_*), where *N* is the constant axial load applied to the column, *f_c_* is the design axial compressive strength of concrete, and *A_c_* is the gross cross-sectional area of the column. Accordingly, axial compression ratios of 0.2, 0.3, and 0.4 corresponded to prescribed axial loads of 257.4, 386.1, and 514.8 kN, respectively. The axial load was applied prior to lateral loading and maintained at the prescribed level throughout the test.

To investigate the influence of pre-existing damage on strengthening effectiveness, selected specimens were subjected to monotonic pre-damage loading prior to strengthening. Previous studies have demonstrated that the structural response of RC beam–column joints progressively deteriorates with increasing lateral deformation and that the pre-existing damage condition can significantly influence the subsequent response of repaired or strengthened joints [22,23,24]. In particular, Melo et al. [23] reported that, for exterior RC beam–column joints tested under monotonic lateral loading, yielding occurred at drift ratios of approximately 0.58–0.66%, while the peak lateral resistance was reached at drift ratios of approximately 1.3–1.7%. Owaid and Akhaveissy [24] further adopted increasing lateral monotonic loading to introduce controlled pre-damage into exterior RC beam–column joints prior to repair and strengthening. On this basis, drift ratios of 0.75% and 1.5% were selected in the present study as two prescribed pre-damage levels, providing two distinctly separated deformation conditions within the nonlinear response range reported for RC beam–column joints under monotonic loading. For the present specimen geometry, these drift ratios corresponded to prescribed horizontal displacements of 16 mm and 32 mm at the column top, respectively.

**Figure 1:**
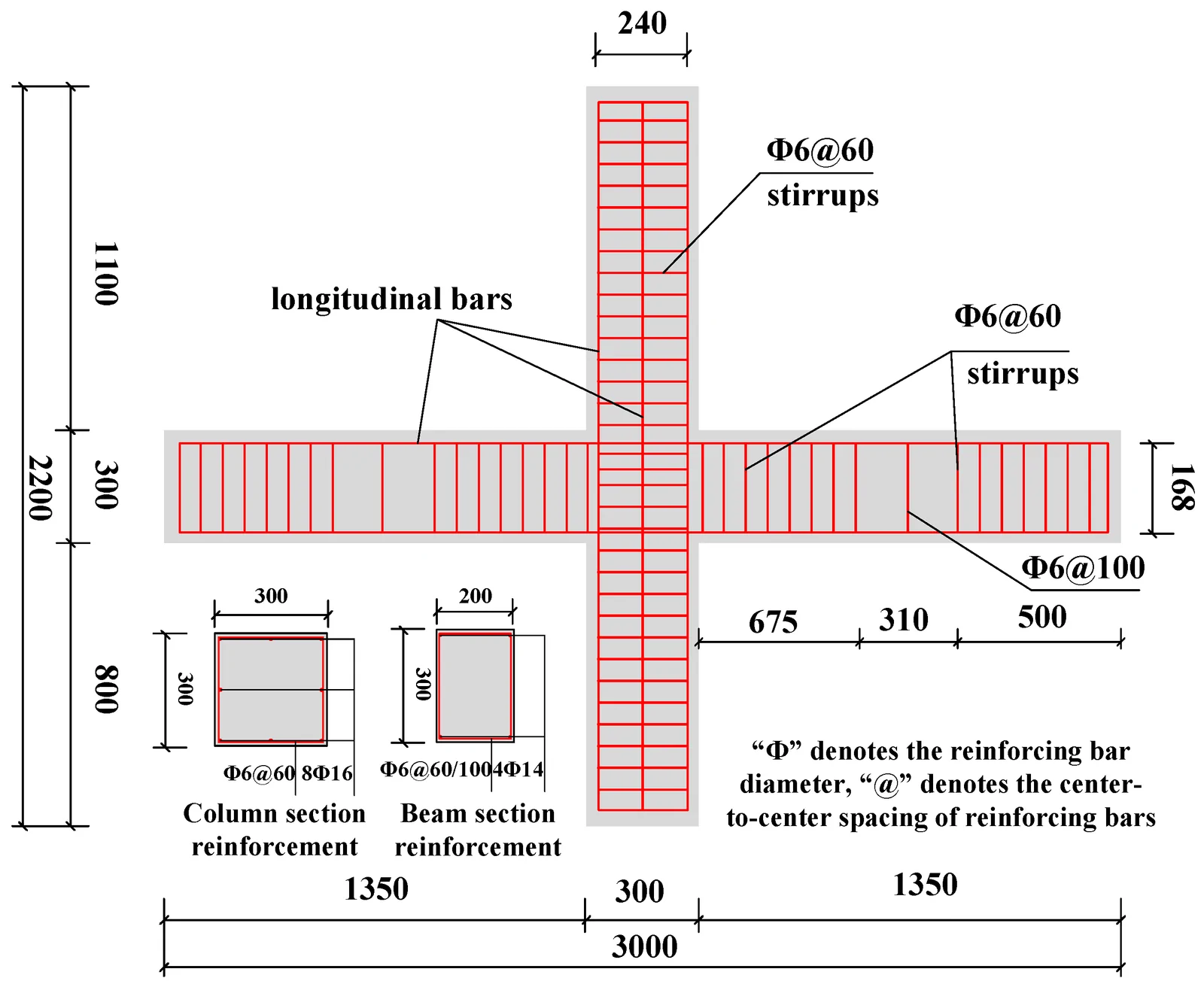
Dimensions and reinforcement details of the original RC beam–column joint (unit: mm).

**Figure 2:**
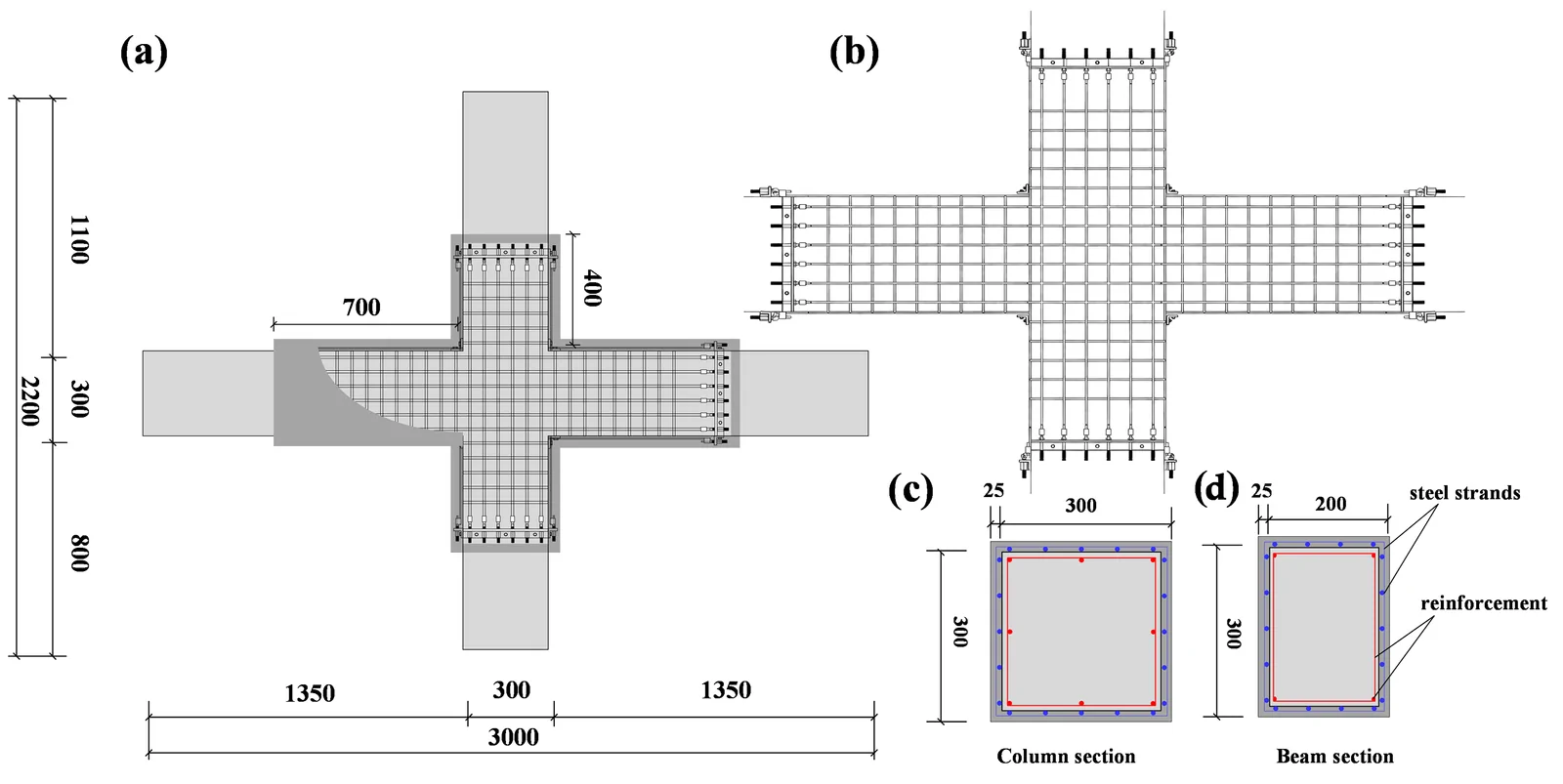
Strengthening configuration and cross-sectional details of the HSSWM–ECC-strengthened joint (unit: mm): (**a**) overall strengthening configuration; (**b**) steel strand mesh layout; (**c**) column cross-section; (**d**) beam cross-section.

**Figure 3:**
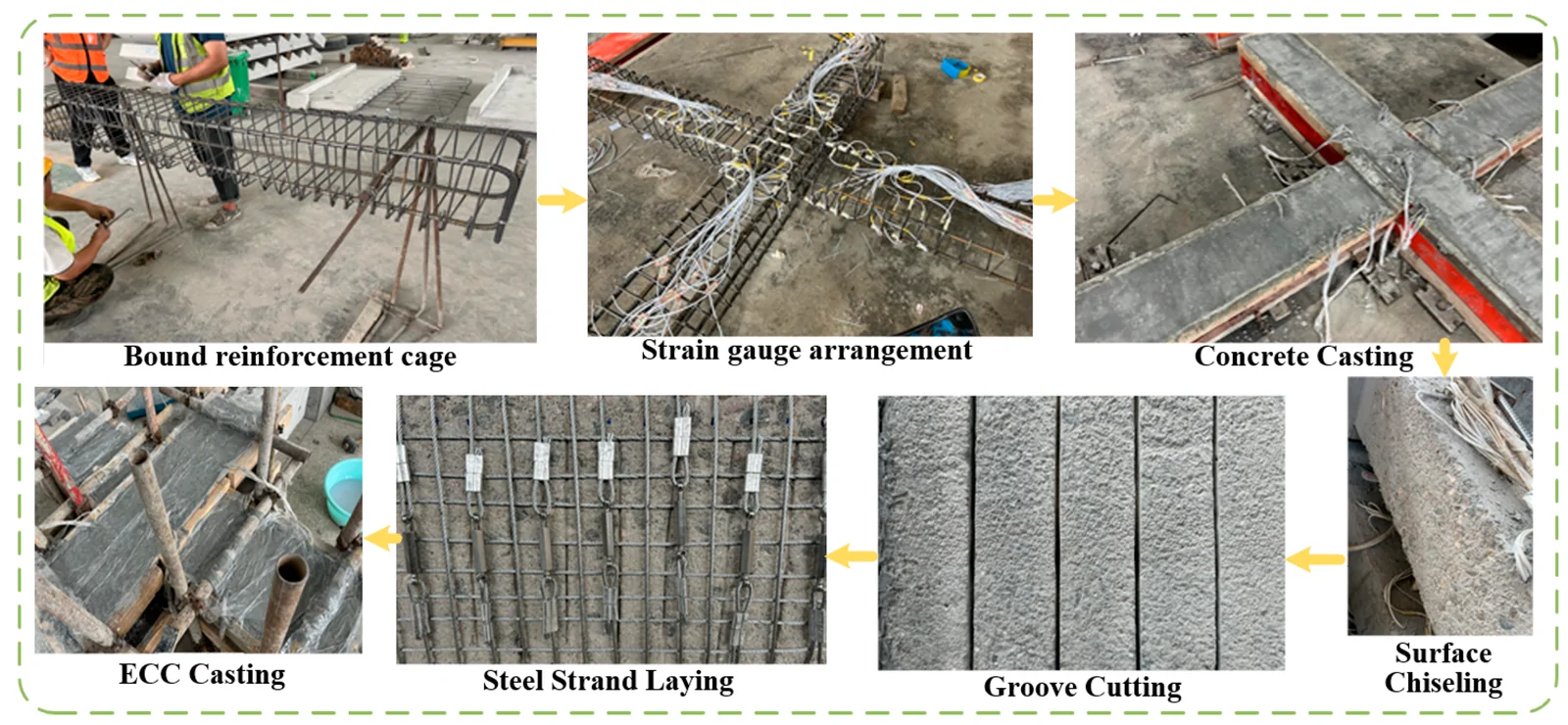
Fabrication and Strengthening Flowchart of Beam–Column Joint.

All specimens had identical beam–column joint dimensions and reinforcement details, as shown in Figure 1. The original RC joints were designed in accordance with GB 50010-2010 [21] and followed the strong-column–weak-beam design philosophy. HRB400 reinforcing bars (Shanghai Zhuyuan Trading Co., Ltd., Shanghai, China) were used for the longitudinal reinforcement, whereas HPB300 bars (Shanghai Zhuyuan Trading Co., Ltd., Shanghai, China) were adopted for the transverse reinforcement. The joint panel was reinforced with 6-mm-diameter closed stirrups at a spacing of 60 mm to provide transverse confinement and joint shear resistance. Accordingly, the specimens were representative of contemporary code-conforming RC beam–column joints. The specified concrete strength grade was C30, and the concrete cover was 25 mm. Considering the anchorage requirements of the steel strands, durability of the strengthening layer, and construction feasibility, together with recommendations reported in the literature [25], the thickness of the strengthening layer was set to 30 mm. The strengthening configuration is illustrated in Figure 2. To enhance composite action between the steel strand mesh and the existing structure and to mitigate premature end debonding and anchorage failure, the strengthening layer was fully wrapped and anchored around the beam and column end regions, thereby improving the anchorage performance of the steel strands embedded within the strengthening layer. The fabrication and strengthening procedure for the RC beam–column joint specimens is presented in Figure 3.

### 2.2. Material Properties

**Figure 4:**
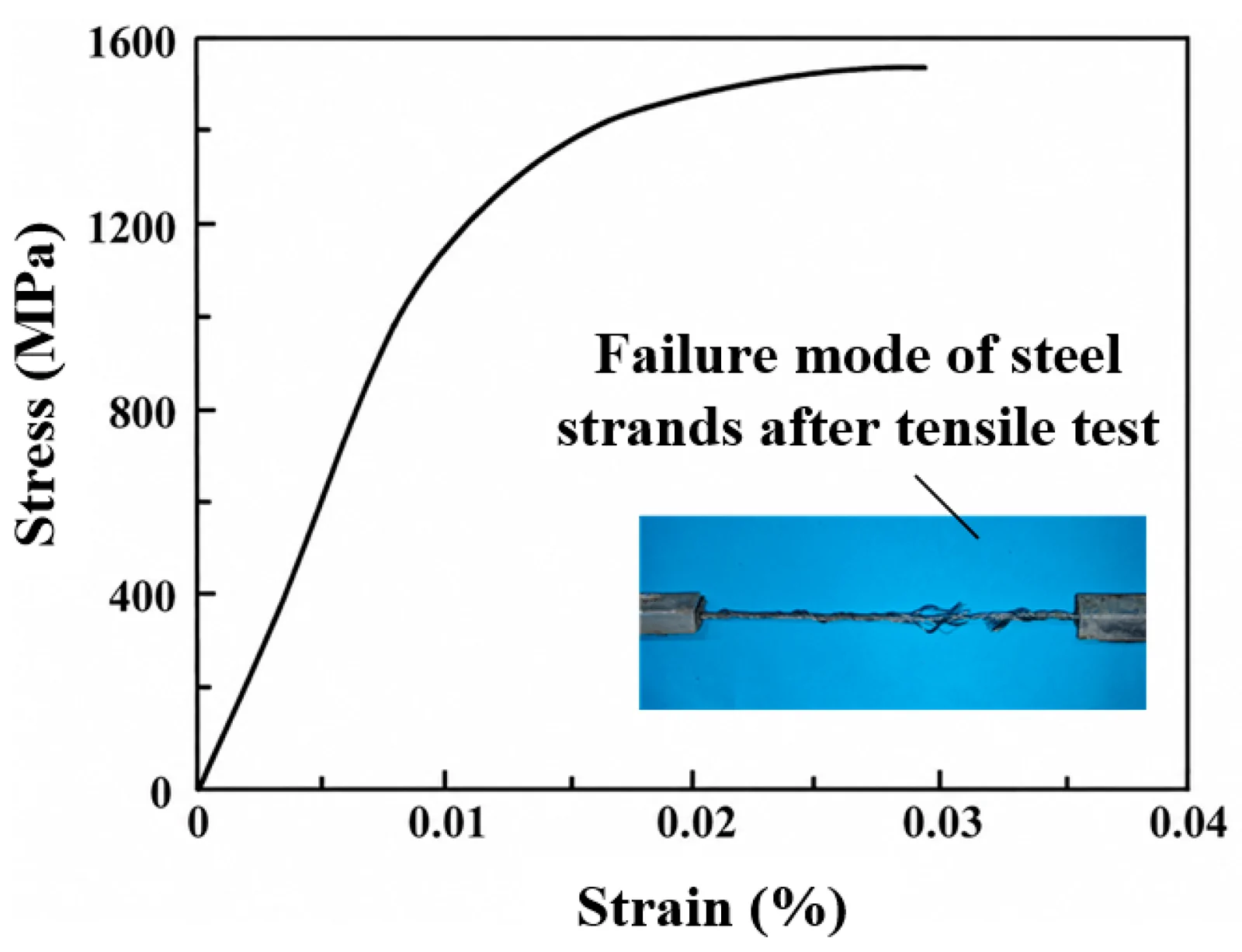
Tensile Stress–Strain Curve of Steel Strand.

**Figure 5:**
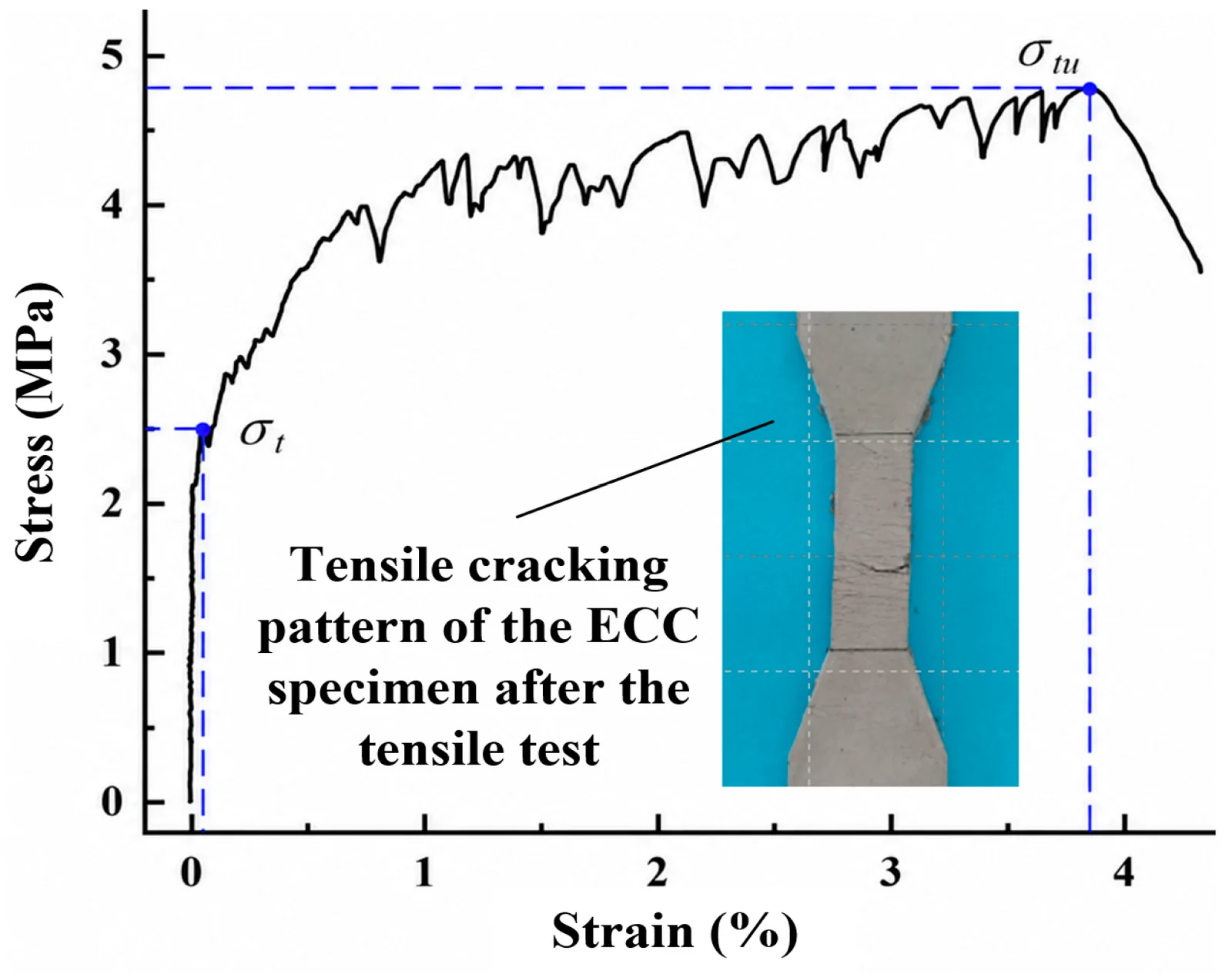
Typical tensile stress–strain curves of ECC.

The main mechanical properties of the test materials are listed in Table 2. The tensile stress–strain response and typical failure mode of the steel strand are shown in Figure 4. The ECC mixture adopted in this study was based on a previously established formulation developed in our research group. The mixture consisted of ordinary Portland cement (P.O. 42.5, Tianrui Group Co., Ltd., Zhengzhou, China), fine sand (Zhengzhou Jingfeng Wear-resistant Material Co., Ltd., Zhengzhou, China), Class I fly ash (Luoyang Haohua Plastic Co., Ltd., Luoyang, China), silica fume (Henan Yuanheng Environmental Protection Engineering Co., Ltd., Zhengzhou, China) with a maximum particle size of 74 μm, water, polyvinyl alcohol (PVA) fibers (Kuraray Co., Ltd., Okayama, Japan), a polycarboxylate-based high-range water-reducing admixture (Shanxi Feike New Material Technology Co., Ltd., Yuncheng, China), and a viscosity-modifying agent (Hebei Shuangniu Building Material Cellulose Co., Ltd., Jinzhou, China). The mixture design was intended to provide the tensile strain-hardening and multiple-cracking characteristics required for the strengthening layer while maintaining adequate workability for casting around the densely distributed steel strand mesh. Cement, fly ash, and silica fume constituted the binder system; fine sand was used as the aggregate phase, and the water and chemical admixtures were proportioned to provide the required matrix consistency and fiber dispersion. The water-to-binder ratio was 0.25, and the PVA fiber content was 2.0% by volume. The detailed mass proportions are given in Table 3. The PVA fibers had a length of 12 mm, a diameter of 39 μm, a tensile strength of 1620 MPa, and an elastic modulus of 42.8 GPa. After casting, the ECC strengthening layers were demolded after 3 days and water-cured twice daily at room temperature until 28 days. ECC specimens used for material characterization were cured under the same conditions as the strengthened specimens. For each ECC batch, three 70.7 mm × 70.7 mm × 70.7 mm cubic specimens were tested in compression at a loading rate of 0.5 kN/s, while dog-bone specimens were subjected to direct tensile tests at 28 days under displacement control at a loading rate of 0.5 mm/min in accordance with JC/T 2461-2018 [26]. Deformation during the tensile tests was measured using displacement transducers installed on both sides of the specimens. Based on the 21 tensile test results, the mean tensile strength and ultimate tensile strain were 4.24 MPa and 3.83%, respectively, with corresponding coefficients of variation of 3.28% and 1.06%. A representative tensile stress–strain response and the corresponding cracking pattern of the ECC specimen are shown in Figure 5. The batch-average compressive strength ranged from 43.33 to 48.87 MPa, with an overall mean of 46.20 MPa and a batch-to-batch coefficient of variation of 4.03%.

### 2.3. Test Setup and Loading Protocol

**Figure 6:**
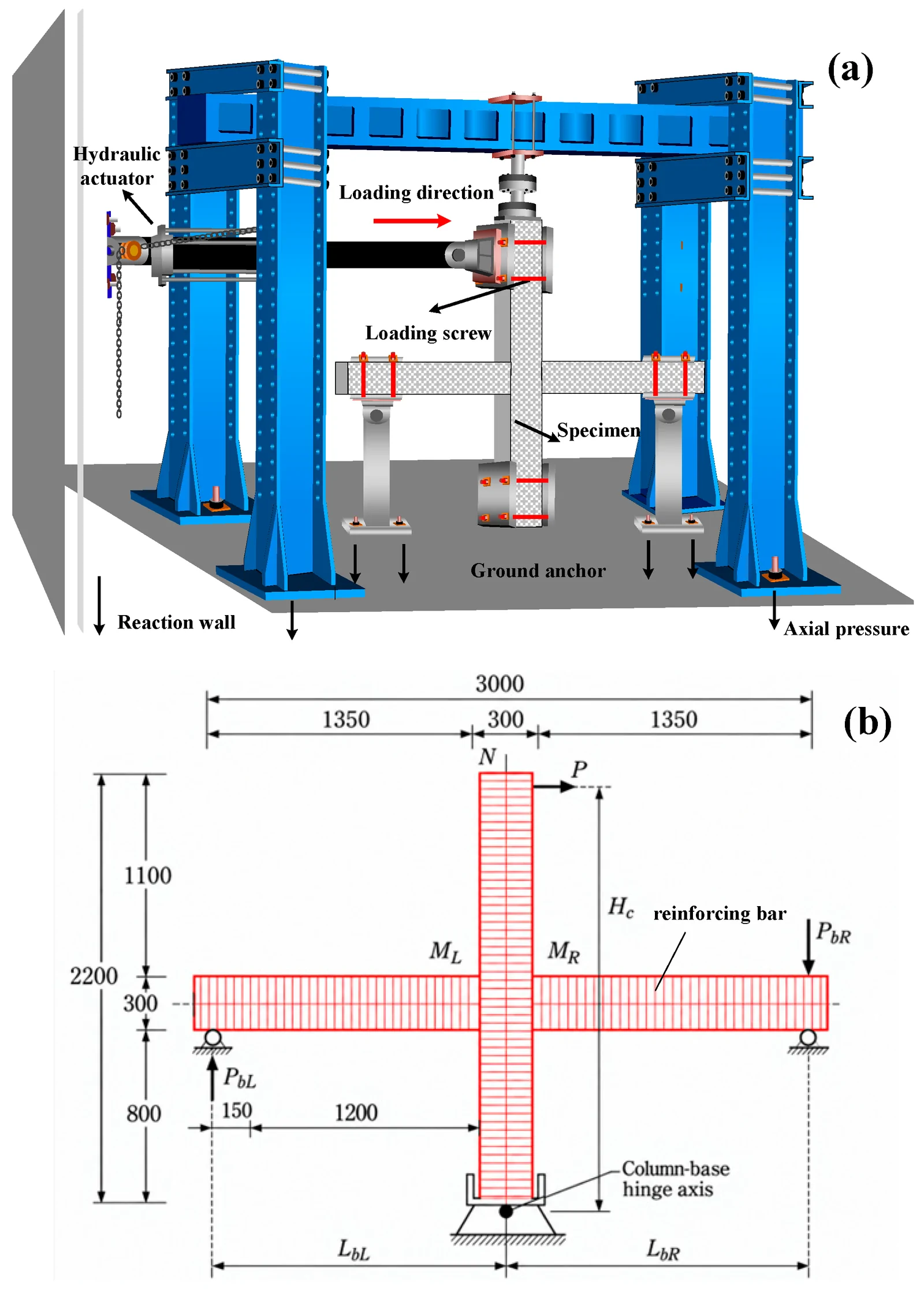
Experimental setup and loading configuration: (**a**) photograph of the test setup; (**b**) free-body diagram and schematic representation of the loading and support conditions. (unit: mm). Note: *N* denotes the axial load applied to the column; *P* is the horizontal load applied at the column top; *H_c_* is the vertical distance from the line of action of *P* to the column-base hinge axis; *P_bL_* and *P_bR_* are the vertical reactions at the left and right beam-end supports, respectively; *L_bL_* and *L_bR_* are the corresponding horizontal distances from the beam-end supports to the column-base hinge axis; and *M_L_* and *M_R_* are the bending moments at the left and right beam-end critical sections, respectively.

The test setup is shown in Figure 6. Before lateral loading, the prescribed axial load, corresponding to the target axial compression ratio defined in Section 2.1, was applied at the column top using a vertical hydraulic jack (Wanding Machinery Tools Co., Ltd., Taizhou, China). After the target axial load was reached, it was maintained at the prescribed level throughout the subsequent horizontal loading process, such that the axial compression ratio remained unchanged during the test. Horizontal loading was then applied at the column top using an electrohydraulic servo actuator (Jinan Docer Testing Machine Technology Co., Ltd., Jinan, China). Following the loading schemes adopted in previous studies on RC beam–column joints [23,24] and HSSWM–ECC-strengthened RC members [17,18], a monotonic displacement-controlled protocol was employed. The monotonic loading scheme was consistent with the primary focus of the present study on the evolution of beam-end flexural response and ultimate resistance, which provided the experimental basis for the sectional flexural-capacity model developed herein. By excluding load reversals, the loading protocol allowed the progression of cracking, reinforcement yielding, mobilization of the HSSWM–ECC strengthening layer, and development of peak resistance to be examined without the additional influence of cyclic stiffness and strength degradation or cumulative damage. Prior to yielding, the lateral displacement was increased in increments of 2 mm. Once yielding was identified, the measured yield displacement, Δ*y*, was adopted as the displacement increment for the subsequent loading stages. Loading was continued until the resistance decreased to 70% of the peak value to capture the post-peak response. For the pre-damaged specimens, monotonic pre-loading was first applied under the prescribed constant axial load until the column-top horizontal displacement reached 16 or 32 mm, corresponding to drift ratios of 0.75% and 1.50%, respectively, as defined in Section 2.1. The specimens were then unloaded in the lateral direction. Following HSSWM–ECC strengthening and 28 days of curing, the specimens were subjected to the formal monotonic loading test using the same loading protocol as that adopted for the initially undamaged specimens.

## 3. Test Results

### 3.1. Failure Modes

**Figure 7:**
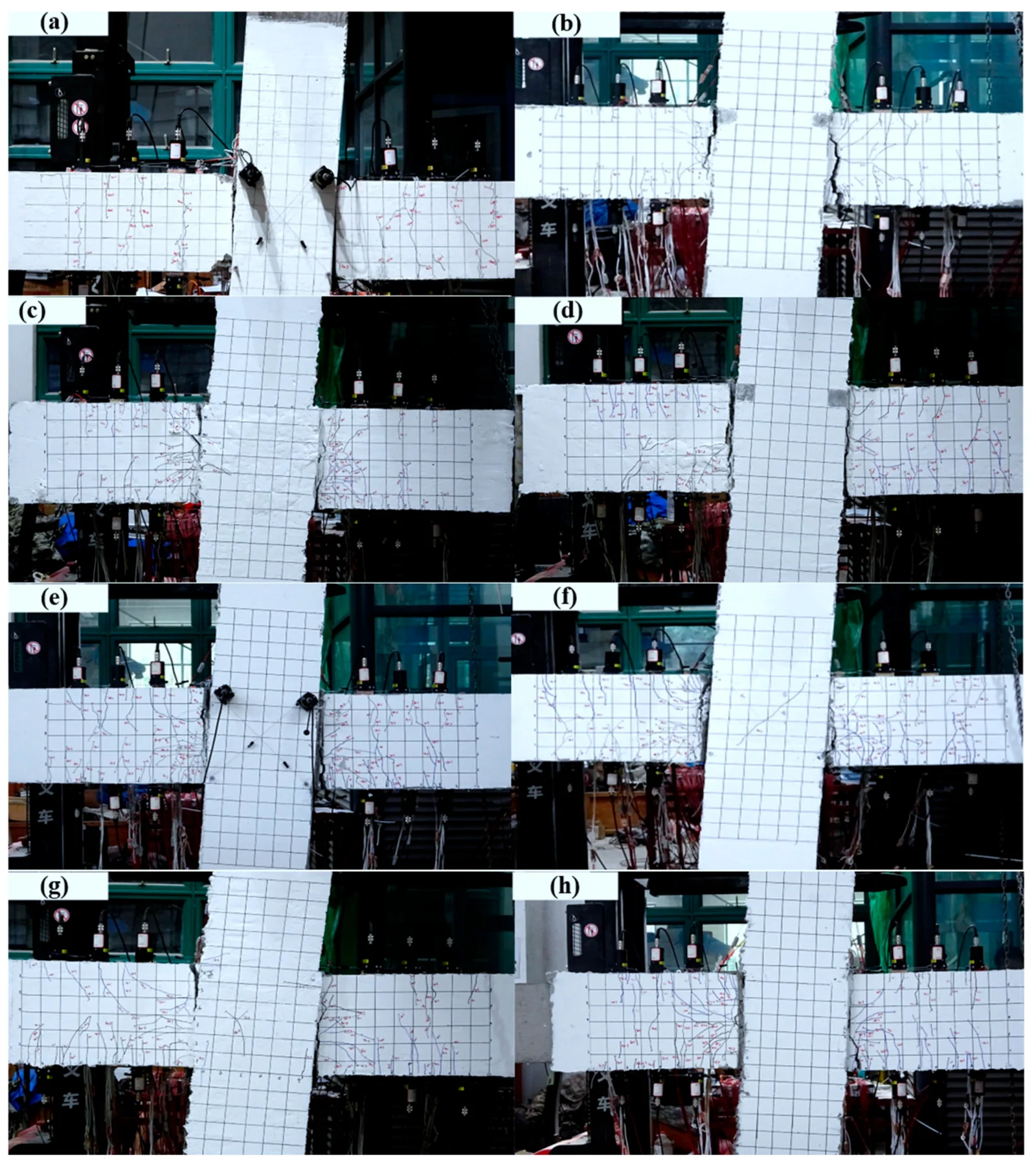
Failure patterns of the tested beam–column joint specimens: (**a**) S-0-0-0.3; (**b**) S-0-50-0.3; (**c**) S-0-30-0.3; (**d**) S-0-70-0.3; (**e**) S-16-50-0.3; (**f**) S-32-50-0.3; (**g**) S-0-50-0.2; (**h**) S-0-50-0.4.

Based on the observed cracking behavior and failure characteristics, the eight beam–column joint specimens exhibited three representative crack-development and failure modes, as illustrated in Figure 7.

Unstrengthened beam–column joint specimen S-0-0-0.3: At the initial loading stage, vertical flexural cracks first developed in the tensile zone of the beam end. With increasing displacement, both the number and width of the cracks increased progressively, with cracking gradually concentrating near the beam–column interface. After yielding of the longitudinal tensile reinforcement in the beam, the formation of new cracks became less pronounced, while the existing cracks widened rapidly, accompanied by a marked increase in beam-end deformation. At the later loading stage, crushing and spalling of concrete occurred in the compression zone of the beam end, and a dominant crack developed along the beam–column interface. The specimen ultimately failed in beam-end flexure, while no significant damage was observed in either the column or the joint core, as shown in Figure 7a.

Undamaged strengthened beam–column joint specimens S-0-50-0.3, S-0-30-0.3, S-0-70-0.3, S-0-50-0.2, and S-0-50-0.4: At the initial loading stage, cracking first occurred in the ECC within the tensile zone of the beam end, followed by the progressive formation of numerous fine vertical and inclined cracks. These cracks were relatively short and distributed uniformly over the strengthened region. As loading progressed, cracks near the beam–column interface gradually widened; however, crack localization was effectively suppressed by the multiple-cracking capacity of ECC and the tensile restraint provided by the steel strand mesh. After yielding of the beam longitudinal reinforcement, both beam-end deformation and the width of the dominant crack increased rapidly, while distributed cracking within the ECC continued to develop. At the later loading stage, local bulging of the ECC occurred near the beam–column interface, accompanied by rupture of several steel strands. Nevertheless, no extensive debonding of the strengthening layer was observed, and the specimens ultimately failed in beam-end flexure, as shown in Figure 7b–d,g–h.

**Figure 8:**
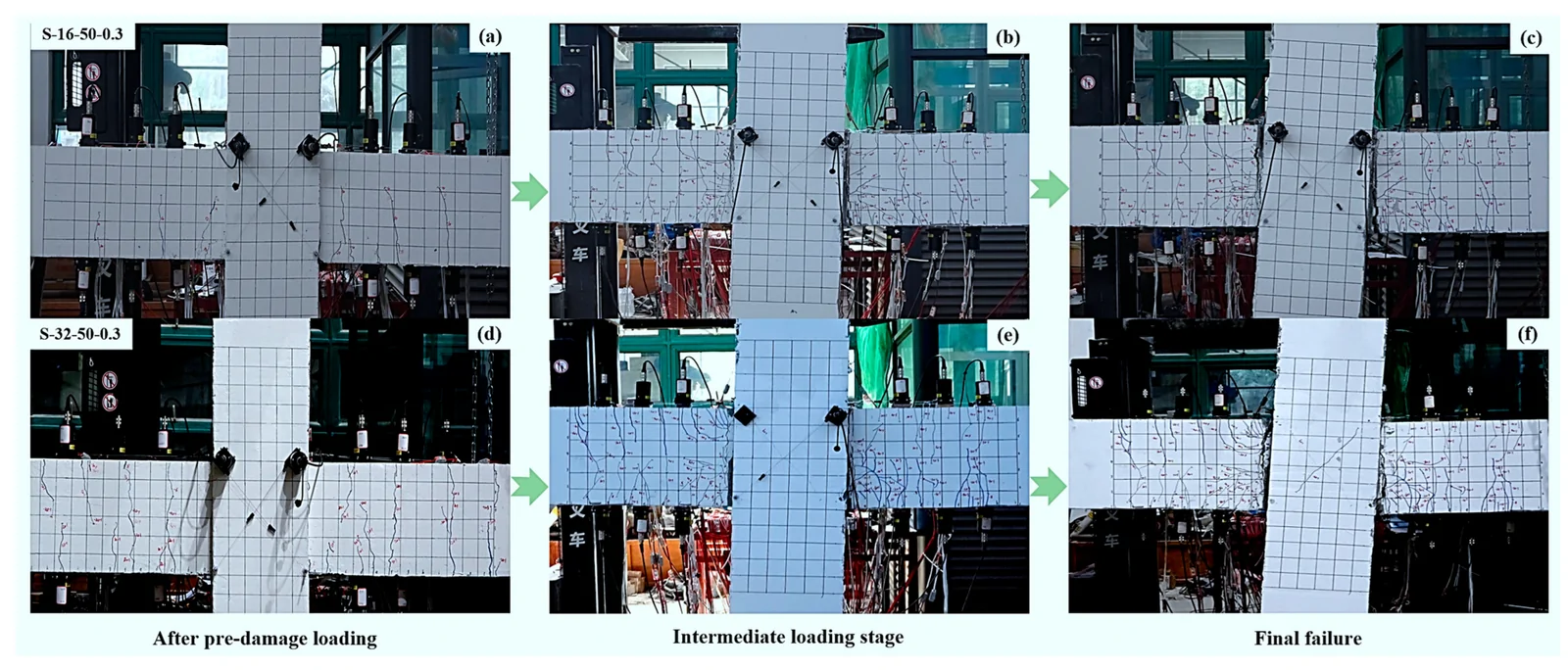
Representative crack evolution of the pre-damaged specimens: (**a**–**c**) S-16-50-0.3; (**d**–**f**) S-32-50-0.3.

Pre-damaged strengthened beam–column joint specimens S-16-50-0.3 and S-32-50-0.3: At the initial stage of the formal loading test, cracks preferentially formed in the strengthening layer at locations corresponding to the pre-existing damage cracks. Subsequently, additional cracks developed within the ECC and progressively connected with cracks in the original concrete substrate. Following yielding of the beam longitudinal reinforcement, the width of the pre-existing dominant cracks and the beam-end deformation increased rapidly, whereas the ECC continued to exhibit a fine and distributed cracking pattern. With increasing pre-damage level, c racking occurred at an earlier stage of the subsequent loading process, while crack propagation and localized damage became more pronounced. In particular, specimen S-32-50-0.3 exhibited more pronounced crack development than S-16-50-0.3. Representative crack evolution of the two pre-damaged specimens is presented in Figure 8, including the condition after pre-damage loading, an intermediate stage of the post-strengthening loading, and the final failure state. At the later loading stage, both specimens showed pronounced crack opening near the beam–column interface and localized damage to the strengthening layer. Nevertheless, the column and joint core remained largely intact, and both specimens ultimately failed in beam-end flexure.

### 3.2. Load–Displacement Response

**Figure 9:**
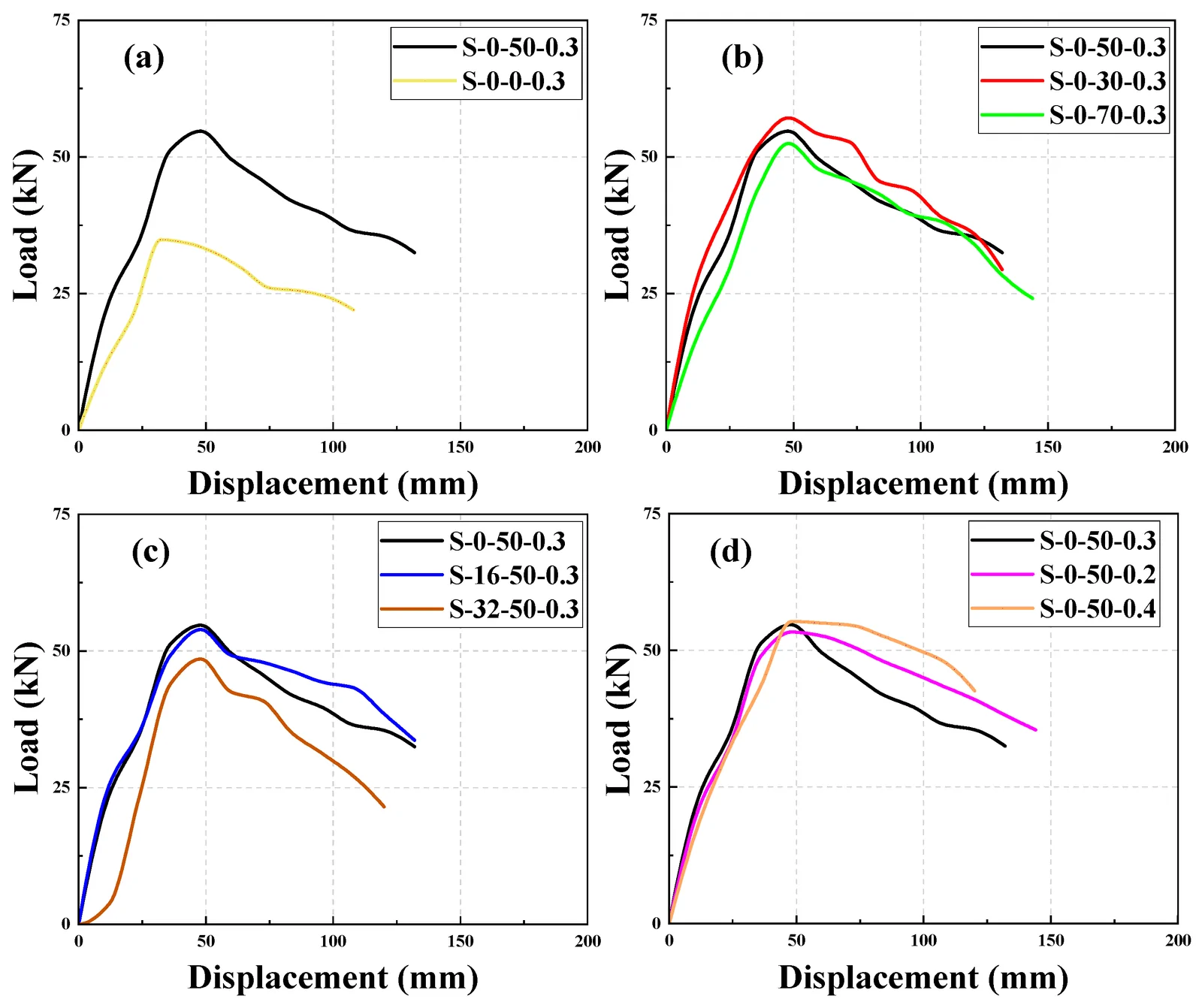
Load–displacement skeleton curves under different test parameters. (**a**) Unstrengthened and strengthened specimens. (**b**) Varied steel strand spacing. (**c**) Different pre-damage levels. (**d**) Different axial compression ratios.

Figure 9 presents the load–displacement curves of all specimens. Figure 9a compares the load–displacement responses of the unstrengthened specimen S-0-0-0.3 and the reference strengthened specimen S-0-50-0.3. Overall, the responses can be divided into four stages: approximately linear loading, progressive stiffness degradation, attainment of the peak load, and post-peak softening. The unstrengthened specimen S-0-0-0.3 exhibited relatively low initial stiffness and rapid post-peak strength degradation. With increasing displacement, progressive cracking reduced the effective stiffness of the concrete section, while yielding of the longitudinal reinforcement further accelerated stiffness degradation. In contrast, the strengthened specimens exhibited markedly higher initial stiffness, peak load, and post-peak resistance, with peak-load increases of approximately 39.24–63.88%. In particular, the peak load of S-0-50-0.3 was 56.94% higher than that of S-0-0-0.3, indicating that the HSSWM–ECC layer effectively increased the tensile resistance of the beam-end section and enhanced its flexural capacity. Moreover, the strengthened specimens exhibited a more gradual post-peak decline, suggesting that the multiple-cracking behavior of ECC delayed crack localization and damage progression.

The effect of steel strand spacing is shown in Figure 9b. The peak loads of S-0-50-0.3, S-0-30-0.3, and S-0-70-0.3 were 54.71, 57.13, and 52.48 kN, respectively. Reducing the strand spacing from 70 to 30 mm increased the peak load by approximately 8.86%. Compared with S-0-0-0.3, the corresponding increases in load-carrying capacity were 56.94%, 63.88%, and 50.55%, respectively. This trend indicates that a smaller strand spacing increases the effective tensile area per unit width of the strengthening layer, thereby increasing the additional tensile force carried by the steel strand mesh and improving the flexural resistance of the beam-end section.

The effect of pre-damage level is shown in Figure 9c. Specimen S-16-50-0.3 reached a peak load of 53.92 kN, only 1.44% lower than that of the undamaged strengthened specimen S-0-50-0.3, indicating that HSSWM–ECC strengthening can largely compensate for the capacity reduction associated with the lower pre-damage level. With increasing pre-damage level, however, specimen S-32-50-0.3 exhibited reductions in initial stiffness and post-peak resistance, with its peak load decreasing to 48.54 kN, 11.28% lower than that of S-0-50-0.3. This reduction is mainly attributed to pre-existing cracking, concrete damage, and residual deformation, which weakened the effective contribution of the original RC section and reduced the composite action between the existing member and the strengthening layer. Therefore, within the investigated range, the effectiveness of HSSWM–ECC strengthening decreases as the pre-damage level increases.

The effect of axial compression ratio is shown in Figure 9d. Within the investigated range of 0.2–0.4, the variation in peak load remained within 3.64%, indicating a relatively low sensitivity of peak flexural resistance to axial compression ratio. By contrast, the post-peak response exhibited a more pronounced dependence on axial compression. Specimen S-0-50-0.2 showed a larger ultimate displacement than S-0-50-0.3 and S-0-50-0.4. As the axial compression ratio increased from 0.2 to 0.4, second-order *P*–*Δ* effects became more pronounced, accompanied by accelerated crack propagation and steeper post-peak strength degradation. These results suggest that, within the tested range, axial compression ratio exerted a greater influence on post-peak deformation capacity than on peak flexural resistance.

### 3.3. Beam-End Flexural Mechanism

Based on the experimental responses and failure characteristics, the flexural behavior of HSSWM–ECC-strengthened beam ends can be divided into four stages: composite action, post-cracking force redistribution, yielding of the longitudinal reinforcement, and ultimate failure. Initially, the original RC section and the strengthening layer deformed compatibly and jointly resisted the applied forces. After cracking in the tensile zone, the concrete tensile contribution decreased rapidly, and the tensile force was transferred mainly to the beam longitudinal reinforcement and HSSWM–ECC layer. Qin et al. [14] showed that the multiple-cracking behavior of ECC suppresses crack localization and promotes coordinated action within the section, while Tian et al. [15] demonstrated the importance of interfacial bond to crack development and composite behavior. In the present tests, fiber bridging redistributed tensile strain, the steel strand mesh provided a continuous tensile path across cracks, and surface roughening, grooving, and wrap-around anchorage ensured effective interfacial force transfer.

With increasing load, yielding of the beam longitudinal reinforcement caused a marked reduction in sectional stiffness and increased mobilization of the strengthening system. In all specimens, the beam longitudinal reinforcement yielded, whereas the joint-core transverse reinforcement generally remained below yield, consistent with the absence of premature joint-panel shear failure and indicating that the nonlinear response was governed primarily by beam-end flexure. The observed flexure-dominated response provides the experimental basis for treating the beam-end section as the critical section in the subsequent sectional analysis. Compression was mainly resisted by the original concrete and ECC in compression, while tension was carried by the beam reinforcement, steel strand mesh, and tensile ECC. Li et al. [17] similarly reported that HSSWM–ECC delays crack propagation and enhances flexural capacity. In the present wrap-around configuration, the side strengthening layers also contributed to sectional resistance.

For the pre-damaged specimens, existing cracks and residual deformation produced an initial strain mismatch between the original RC section and the newly added layer, resulting in strain lag during reloading. Wei et al. [19] reported that secondary loading can delay mobilization of the strengthening layer, while Zhao et al. [20] showed that residual strain significantly affects the subsequent response of repaired RC beams. Initial damage therefore both reduces the residual resistance of the original section and delays engagement of the HSSWM–ECC system. This explains why S-16-50-0.3 exhibited a peak capacity close to that of S-0-50-0.3, whereas S-32-50-0.3 showed a more pronounced reduction, and provides the basis for introducing a damage reduction parameter in the subsequent model.

After peak load, the dominant crack at the beam–column interface continued to widen, accompanied by increasing compression-zone damage, local ECC bulging, and rupture of several steel strands. No extensive debonding or anchorage failure occurred before peak load, confirming the effectiveness of the adopted interface treatment and wrap-around anchorage. All specimens ultimately failed in beam-end flexure, while the column and joint core remained largely intact. Similar behavior was reported by Suryanto et al. [12], indicating that ECC can suppress joint-core damage and localize nonlinear deformation at the adjacent beam end. The present specimens were intentionally designed to develop a beam-end flexural failure mode, and the joint panels remained essentially intact during both the pre-loading and post-strengthening loading stages. Accordingly, the experimental evidence obtained herein primarily reflects the effectiveness of HSSWM–ECC in restoring and enhancing the flexural response of joints with damage concentrated at the beam ends. Previous studies have shown that repair or strengthening interventions applied to the joint region can improve the response of damaged or deficient RC beam–column joints [22,24,27,28], while the use of fiber-reinforced cementitious materials can enhance crack control and preserve joint integrity [11,12,29]. For joints governed primarily by joint-panel damage, the wrap-around HSSWM–ECC configuration may provide additional confinement and distributed tensile resistance; however, this failure condition was not examined in the present study and requires dedicated experimental investigation. Overall, HSSWM–ECC enhances beam-end flexural resistance by increasing tensile capacity, controlling crack localization, and maintaining effective interfacial force transfer, providing the mechanical basis for the subsequent capacity model.

Shrinkage of the ECC strengthening layer may affect the interfacial compatibility of the HSSWM–ECC strengthening system. Because shrinkage of the newly applied ECC is restrained by the existing RC substrate and the embedded steel strand mesh, self-equilibrated stresses may develop within the strengthening layer, accompanied by additional interfacial stresses at the ECC–concrete interface. Such restraint effects may modify the initial strain state of the composite section and, when sufficiently pronounced, impair deformation compatibility or promote localized interfacial cracking, thereby reducing the efficiency of stress transfer. In the present specimens, no extensive interfacial debonding was observed before peak resistance, indicating that shrinkage-related effects were not dominant in the short-term flexural response under the investigated conditions. Nevertheless, shrinkage strain and its long-term influence on interfacial bond and deformation compatibility were not directly quantified. Further investigation is therefore required to assess the sustained composite action and long-term performance of the strengthening system.

## 4. Analytical Model for Beam-End Flexural Capacity

Existing studies on the flexural capacity of RC members strengthened with ECC or ECC-based composites have focused mainly on isolated beams. Most analytical models assume adequate interfacial bonding and compatible deformation between the strengthening layer and the original section, and primarily account for the contribution of the tensile strengthening layer [14,15,17]. Recent studies have begun to consider strain lag induced by secondary loading and residual strain after unloading [19,20]; however, the combined effects of pre-existing damage and side strengthening in wrap-around-strengthened beam–column joints remain insufficiently understood. In the present specimens, pre-damage loading produced cracking, stiffness degradation, and residual deformation at the beam end, preventing full strain compatibility between the original RC section and the subsequently added HSSWM–ECC layer during early reloading. Moreover, the wrap-around configuration allows the side HSSWM–ECC layers to extend across both the tensile and compressive zones and participate in sectional force transfer, so their flexural contribution cannot be neglected. Accordingly, a baseline beam-end flexural capacity model is first established for the undamaged condition, followed by incorporation of the strain-lag associated with the pre-damage history and the contribution of the side strengthening layers, leading to a calculation method applicable to damaged HSSWM–ECC-strengthened beam–column joints.

### 4.1. Critical Section and Experimental Moment

The test results showed that cracking and nonlinear deformation in all specimens were primarily concentrated in the beam-end regions adjacent to the column faces. The unstrengthened specimen ultimately exhibited concrete crushing and spalling in the beam-end compression zone, whereas the strengthened specimens developed a dominant crack at the beam–column interface, accompanied by local ECC bulging and rupture of several steel strands. In all cases, the columns and joint cores remained essentially intact. In addition, the strains in the beam longitudinal reinforcement exceeded the corresponding yield strains, while the stirrups in the joint core generally remained below yield. These observations indicate that the nonlinear response of the specimens was governed primarily by flexural deformation at the beam ends. Accordingly, the beam-end section at the beam–column interface was selected as the critical section for flexural-capacity evaluation.

The specimen was idealized as the subassembly of a frame bounded by the inflection points of the beams and column, where the bending moments are assumed to vanish. The corresponding free-body diagram is shown in Figure 6b. The column base and the two beam-end supports were therefore represented by hinges. The lateral load applied at the column top is denoted by *P*, and *H_c_* is the vertical distance between its line of action and the hinge axis at the column base. The vertical reactions measured at the left and right beam-end supports are denoted by *P_bL_* and *P_bR_*, respectively, while *L_bL_* and *L_bR_* are their corresponding horizontal lever arms with respect to the column-base hinge axis. Taking moments about the column-base hinge gives the following:

(1) $$P_{bL}L_{bL}+P_{bR}L_{bR}=PH_{c}$$

The critical sections for flexural-capacity evaluation were located at the beam–column interfaces. Denoting the distance from each beam-end support to the corresponding critical section by *L_b_*, the experimentally derived beam-end moments are as follows:

(2) $$M_{L}=P_{bL}L_{b}$$

(3) $$M_{R}=P_{bR}L_{b}$$

For the symmetric test configuration adopted herein, *L_bL_* = *L_bR_* = *L*. Substitution of Equations (2) and (3) into Equation (1) therefore yields the following:

(4) $$M_{L}+M_{R}=P_{bL}L_{b}+P_{bR}L_{b}=\frac{PH_{c}}{L}\times L_{b}$$

Equation (4) establishes the relationship between the externally measured column-top load and the resultant flexural demand at the two critical beam-end sections.

### 4.2. Model Assumptions

A plane-section analysis was adopted to develop the beam-end flexural-capacity model for the HSSWM-ECC-strengthened joints. To facilitate the analytical formulation, the following assumptions were made:

(1) At peak resistance, the specimens reached a drift ratio of approximately 2%, with inelastic deformation predominantly concentrated at the beam end adjacent to the column face. Consistent with the observed yielding of the beam longitudinal reinforcement and the essentially elastic response of the joint-core reinforcement, the critical beam-end section was analyzed on the basis of the plane-sections-remain-plane assumption. The resulting linear strain distribution through the section depth was subsequently used to determine the strains of the constituent materials from sectional compatibility.

(2) After the cracking of the concrete in the tensile region of the beam end, its tensile contribution was considered negligible compared with that of the longitudinal reinforcement and the HSSWM-ECC strengthening layer. Accordingly, the tensile resistance of concrete was neglected in the sectional flexural-capacity analysis, and the resultant tensile force was assumed to be jointly resisted by the original beam longitudinal reinforcement, the ECC in tension, and the steel strand mesh.

(3) Owing to the relatively small cross-sectional area and axial stiffness of the steel strands in the compression zone, their contribution to sectional force equilibrium was considered insignificant. Therefore, the compressive contribution of the steel strands was neglected, and only their tensile contribution was considered in the analysis.

(4) The compressive stress–strain relationship curve of concrete is expressed in Equation (5) [21], *ε*_0_ denotes the compressive strain corresponding to *f_c_* and was taken as 0.002; *ε_cu_* denotes the ultimate compressive strain of concrete and was taken as 0.0033; and n was taken as 2. In addition, *σ_c_* and *f_c_* denote the compressive stress and axial compressive strength of concrete, respectively.

(5) $$\sigma_{c}=\left\{\begin{array}{ll} f_{c}\left[1-{\left(1-\frac{\varepsilon_{c}}{\varepsilon_{0}}\right)}^{n}\right], & \varepsilon_{c}⩽\varepsilon_{0}\\ \sigma_{c}=f_{c}, & \varepsilon_{0}⩽\varepsilon_{c}⩽\varepsilon_{\mathrm{cu}} \end{array}\right.$$

(5) The stress–strain relationship of the reinforcing steel was represented by a bilinear elastic–perfectly plastic model [21], as given in Equation (6):

(6) $$\sigma_{s}=\left\{\begin{array}{ll} E_{s}\varepsilon_{s} & (\varepsilon_{s}<\varepsilon_{y})\\ f_{y} & (\varepsilon_{s}>\varepsilon_{y}) \end{array}\right.$$

where *E_s_* is the elastic modulus of the reinforcing steel, *f_y_* is the yield strength, *ε_y_* = *f_y_*/*E_s_* is the corresponding yield strain, and *ε_s_* denotes the strain of the reinforcing steel.

(6) Both the tensile and compressive contributions of ECC were considered in the analysis. To characterize its compressive stress–strain response, the constitutive model proposed by Zou et al. [30] was adopted, as expressed as follows:

(7) $$\sigma_{ec}=\left\{\begin{array}{ll} \;\left[1.1\frac{\varepsilon_{ec}}{\varepsilon_{ecu}}+0.5{\left(\frac{\varepsilon_{ec}}{\varepsilon_{ecu}}\right)}^{5}-0.6{\left(\frac{\varepsilon_{ec}}{\varepsilon_{ecu}}\right)}^{6}\right]\sigma_{ecu} & \left(\frac{\varepsilon_{ec}}{\varepsilon_{ecu}}\leq 1\right)\\ \;\left[\frac{0.15{\left(\frac{\varepsilon_{ec}}{\varepsilon_{ecu}}\right)}^{2}}{1-2\frac{\varepsilon_{ec}}{\varepsilon_{ecu}}+1.15{\left(\frac{\varepsilon_{ec}}{\varepsilon_{ecu}}\right)}^{2}}\right]\sigma_{ecu} & \left(\frac{\varepsilon_{ec}}{\varepsilon_{ecu}}\geq 1\right) \end{array}\right.$$

where *σ_ec_* and *ε_ec_* denote the compressive stress and the corresponding compressive strain of ECC, respectively; *ε_ecu_* is the ultimate compressive strain of ECC, generally taken as 0.005; and *σ_ecu_* is the compressive stress corresponding to *ε_ecu_*.

For the tensile response of ECC, a commonly used bilinear constitutive model was adopted, expressed as follows:

(8) $$\sigma_{et}=\left\{\begin{array}{ll} \frac{\sigma_{ecr}}{\varepsilon_{ecr}}\varepsilon_{et} & \left(\varepsilon_{et}\leq \varepsilon_{ecr}\right)\\ \sigma_{ecr}+\frac{\sigma_{etu}-\sigma_{ecr}}{\varepsilon_{etu}-\varepsilon_{ecr}}\left(\varepsilon_{et}-\varepsilon_{ecr}\right) & \left(\varepsilon_{ecr}\leq \varepsilon_{et}\leq \varepsilon_{etu}\right) \end{array}\right.$$

where *σ_et_* and *ε_et_* denote the tensile stress and the corresponding tensile strain of ECC, respectively; *σ_ecr_* and *ε_ecr_* are the cracking stress and the corresponding cracking strain; and *σ_etu_* and *ε_etu_* are the ultimate tensile stress and the corresponding ultimate tensile strain, respectively.

(7) The tensile constitutive relationship of the high-strength steel strands was determined based on the results reported by Wang et al. [31] and the material test data obtained in this study. Since the stress–strain response of the high-strength steel strands exhibited no distinct yield plateau, a bilinear model was adopted for simplification, as expressed as follows:

(9) $$\sigma_{sw}=\left\{\begin{array}{ll} \frac{\sigma_{swy}}{\varepsilon_{swy}}\varepsilon_{sw} & \left(\varepsilon_{sw}\leq \varepsilon_{swy}\right)\\ \sigma_{swy}+\frac{\sigma_{swu}-\sigma_{swy}}{\varepsilon_{swu}-\varepsilon_{swy}}(\varepsilon_{sw}-\varepsilon_{swy}) & \left(\varepsilon_{swy}<\varepsilon_{sw}\leq \varepsilon_{swu}\right)\; \end{array}\right.$$

where *σ_sw_* and *ε_sw_* denote the tensile stress and the corresponding tensile strain of the steel strand, respectively; *σ_swy_* and *ε_swy_* are the nominal yield stress and the corresponding yield strain; and *σ_swu_* and *ε_swu_* are the ultimate stress and the corresponding ultimate strain, respectively.

### 4.3. Baseline Flexural-Capacity Model

**Figure 10:**
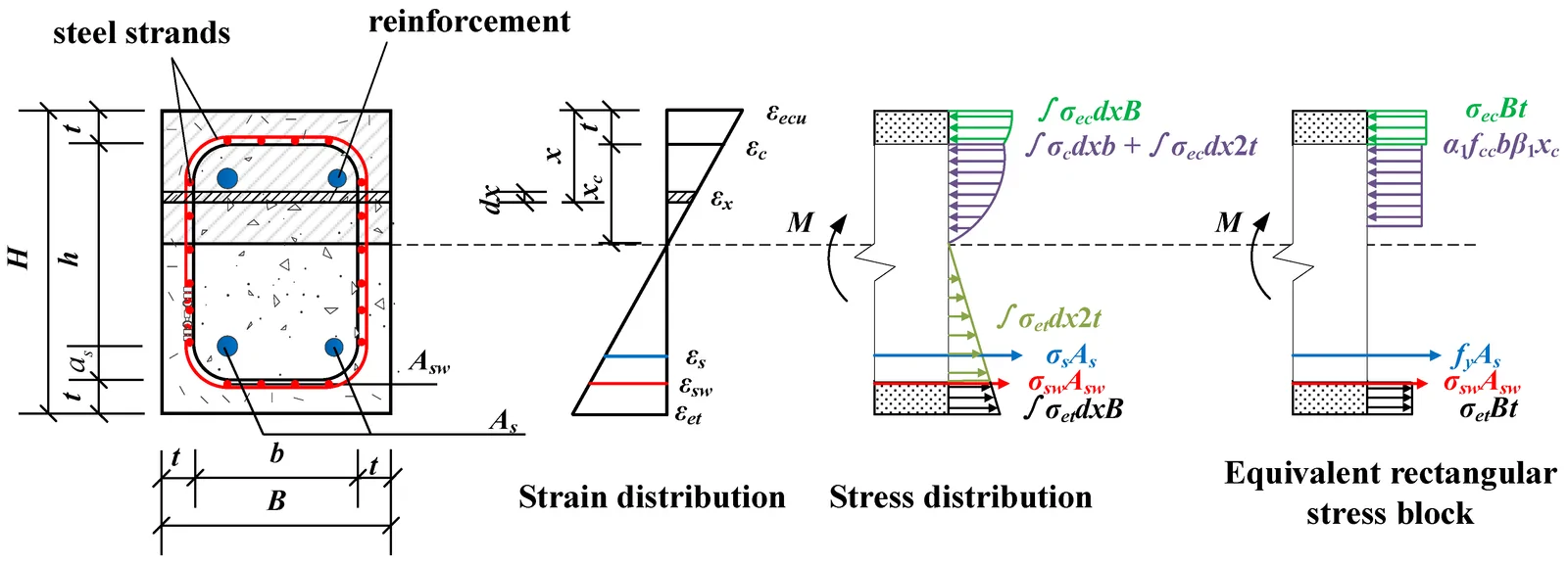
Stress and strain distributions in the beam section of the strengthened joint. Note: *B* and *H* denote the width and overall depth of the strengthened beam section, respectively; *b* and hh denote the width and depth of the original beam section, respectively; *t* is the thickness of the strengthening layer; *x_c_* is the depth of the neutral axis measured from the extreme compression edge of the original concrete; *x* is the sectional depth coordinate, and *dx* is the differential thickness; *A_s_* and *A_sw_* are the cross-sectional areas of the longitudinal tensile reinforcement and the tensile steel strands, respectively; *ε_ecu_* is the ultimate compressive strain of ECC; *ε_ec_*, *ε_et_*, *ε_s_*, and *ε_sw_* are the compressive strain of ECC, tensile strain of ECC, tensile strain of the longitudinal reinforcement, and tensile strain of the steel strands, respectively; *σ_ec_*, *σ_et_*, and *σ_sw_* are the compressive stress of ECC, tensile stress of ECC, and tensile stress of the steel strands, respectively; *α*_1_ and *β*_1_ are the stress and compression-zone depth modification factors for the equivalent rectangular concrete stress block; and *M* is the flexural moment of the critical beam-end section.

During the tests, good composite action was maintained between the HSSWM-ECC strengthening layer and the existing concrete, with no debonding failure observed. The experimental observations further showed that, at the peak load, the longitudinal tensile reinforcement at the beam ends had yielded, and rupture of the steel strands occurred after crushing of the concrete in the compression zone, indicating that the tensile capacity of the HSSWM-ECC strengthening layer had been effectively mobilized. Based on the assumptions described in Section 4.2, the strain and stress distributions over the critical beam-end section of an undamaged strengthened joint can be established, as illustrated in Figure 10.

Under the plane-section assumption, the resulting sectional stress distribution is nonlinear. To simplify the analysis, the nonlinear compressive stress distribution in the original concrete was replaced by an equivalent rectangular stress block while preserving both the resultant compressive force and its line of action. Accordingly, the axial force equilibrium of the critical section can be expressed as follows:

(10) $$\sigma_{ec}Bt+\alpha_{1}f_{c}b\beta_{1}x_{c}=f_{y}A_{s}+\sigma_{sw}A_{sw}+\sigma_{et}Bt$$

Taking moments about the bottom edge of the strengthened beam section, the beam-end flexural capacity can be obtained from the moment equilibrium condition as follows:

(11) $$M=\sigma_{ec}Bt(H-\frac{1}{2}t)+\alpha_{1}f_{c}b\beta_{1}x_{c}(H-t-\frac{\beta_{1}x_{c}}{2})-f_{y}A_{s}(a_{s}+t)-\sigma_{sw}A_{sw}t-\sigma_{et}B\frac{t^{2}}{2}$$

where *M* is the flexural capacity of the critical beam-end section; *B* and *H* are the width and overall depth of the strengthened beam section, respectively; *b* is the width of the original beam section; *t* is the thickness of the strengthening layer; *f_c_* is the axial compressive strength of the original concrete; *α*_1_ and *β*_1_ are the stress and compression-zone depth modification factors, respectively, for the equivalent rectangular concrete stress block; *x_c_* is the distance from the extreme compression edge of the original concrete to the neutral axis; *A_s_* and *A_sw_* are the cross-sectional areas of the longitudinal tensile reinforcement and the tensile steel strands, respectively; *α_s_* is the distance from the bottom edge of the original beam section to the resultant force of the longitudinal tensile reinforcement; *f_y_* is the yield strength of the longitudinal tensile reinforcement; and *σ_ec_*, *σ_et_*, and *σ_sw_* are the stresses in the ECC in compression, ECC in tension, and tensile steel strands, respectively. For concrete with a strength grade not exceeding C50, *α*_1_ = 1.0 and *β*_1_ = 0.80; for higher-strength concrete, the corresponding values should be determined in accordance with the relevant code provisions.

According to the plane-section assumption, the strain at any position within the section can be expressed as follows:

(12) $$\frac{\varepsilon_{x}}{\varepsilon_{ecu}}=\left|\frac{t+x_{c}-x}{t+x_{c}}\right|$$

In the calculation, the strain *ε_x_* at any location within the section is first determined from the strain distribution based on the plane-section assumption and Equation (12). The compressive strain of the ECC in the compression zone at the peak-load state is then substituted into Equation (12) to determine the strains at the required sectional locations. These strains are subsequently introduced into the corresponding constitutive relationships to obtain the compressive stress of ECC *σ_ec_*, the tensile stress of the steel strands *σ_sw_*, and the tensile stress of ECC *σ_et_*. Finally, Equations (10) and (11) are solved simultaneously to determine the beam-end sectional moment *M*.

### 4.4. Effects of Pre-Damage History and Side Strengthening

The equations established in Section 4.3 are applicable to undamaged strengthened joints. For the pre-damaged specimens, cracking and residual strain remained at the beam ends after unloading, resulting in a certain degree of strain incompatibility between the original RC section and the HSSWM-ECC strengthening layer during reloading. This incompatibility limits the full mobilization of the strengthening layer. Therefore, a damage-related modification is introduced into the baseline model for the undamaged condition. To characterize the difference in stiffness deterioration associated with the prescribed pre-damage histories, a stiffness reduction coefficient, *η*, is introduced as follows:

(13) $$\eta =1-\frac{P_{d}/\Delta_{d}}{P_{cr}/\Delta_{cr}}$$

where *P* and Δ*_d_* denote the load and displacement at the prescribed pre-damage state, respectively, while *P_cr_* and Δ*_cr_* denote the corresponding values at first cracking. The secant stiffness at first cracking was adopted as the reference because it represents the onset of stiffness degradation associated with progressive cracking. Accordingly, *η* quantifies the relative reduction in secant stiffness from the cracking state to the prescribed pre-damage state, with a larger *η* indicating a greater extent of stiffness degradation. Based on Equation (13), the values of *η* for specimens S-16-50-0.3 and S-32-50-0.3 were 0.164 and 0.273, respectively.

On this basis, a stress reduction coefficient *γ* was introduced to account for the reduced stress contribution of the HSSWM-ECC strengthening layer due to the pre-damage history:

(14) γ=1−0.03η

A sensitivity analysis was further performed to examine the influence of the stiffness-reduction parameter *η* on the stress-reduction coefficient *γ*. For 0 ≤ *η* ≤ 0.4, *γ* decreases monotonically from 1.000 to 0.988. In particular, the experimentally determined values of *η* = 0.164 and 0.273 correspond to *γ* = 0.995 and 0.992, respectively. These results indicate that *γ* exhibits relatively low sensitivity to variations in *η* within the investigated range.

In addition, a wrap-around strengthening configuration was adopted in this study, in which the HSSWM-ECC layers along the beam sides extended across both the tensile and compressive regions of the section and participated in force transfer at the beam end. Explicitly accounting for the contribution of these side strengthening layers would substantially increase the complexity of the sectional analysis. Therefore, a side-layer utilization coefficient, *η_t_*, was introduced to incorporate their contribution in a simplified manner.

Comparison of the sectional capacities calculated with and without the beam-side strengthening layers showed that neglecting their contribution reduced the predicted flexural capacity by 1.8–7.1%, with an average reduction of approximately 5% (Table 4). A side-layer utilization coefficient, *η_t_*, was therefore introduced to account for this contribution within the simplified sectional formulation. By comparing trial calculations with the full sectional analysis explicitly accounting for the side layers, *η_t_* = 1.1 was found to provide a conservative approximation for the specimens investigated herein and was adopted in the subsequent calculations. The coefficient *η_t_* modifies only the tensile contribution of the steel strands in the sectional equilibrium formulation.

The strengthening contribution of the HSSWM–ECC system arises from two mechanically distinct components. The ECC matrix participates in both tension and compression, whereas the steel strand mesh primarily contributes through tensile force transfer across the critical beam-end section. In the sectional formulation, these contributions are represented separately by the ECC stress resultants, governed by *σ_et_* and *σ_ec_*, and the tensile force in the steel strands, governed by *σ_sw_A_sw_*. This separation indicates that the increase in flexural resistance cannot be attributed solely to the high-strength steel strands: the ECC matrix contributes directly to sectional resistance while simultaneously providing crack distribution, strain compatibility, and stress transfer to the embedded mesh. Nevertheless, because no ECC-only strengthened specimen was included in the experimental program, the individual experimental contributions of the ECC matrix and the steel strand mesh cannot be isolated directly from the measured peak loads. Their relative contributions are therefore interpreted on the basis of the sectional analysis rather than as independently measured quantities.

After accounting for both the initial damage effect and the contribution of the wrap-around side strengthening layers, the axial force equilibrium equation for the critical beam-end section can be expressed as follows:

(15) $$\gamma \sigma_{ec}Bt+\alpha_{1}f_{c}b\beta_{1}x_{c}=f_{y}A_{s}+\eta_{t}(\gamma \sigma_{sw}A_{sw}+\gamma \sigma_{et}Bt)$$

The corresponding moment equilibrium equation is as follows:

(16) $$M=\gamma \sigma_{ec}Bt(H-\frac{1}{2}t)+\alpha_{1}f_{c}b\beta_{1}x_{c}(H-t-\frac{\beta_{1}x_{c}}{2})-f_{y}A_{s}(a_{s}+t)-\eta_{t}(\gamma \sigma_{sw}A_{sw}t-\gamma \sigma_{et}B\frac{t^{2}}{2})$$

### 4.5. Model Validation

The predictive performance of the proposed analytical model was assessed by comparing the calculated beam-end flexural capacities with the corresponding experimental results, as summarized in Table 5. Here, *P_t_* and *P_c_* denote the experimental and calculated capacities, respectively. The mean value, standard deviation, and coefficient of variation of *P_t_*/*P_c_* were 0.955, 0.015, and 0.016, respectively, and the deviations between the calculated and experimental capacities remained within 7%. The overall agreement indicates that the proposed formulation adequately captures the beam-end flexural resistance of the strengthened joints within the investigated parameter range. Nevertheless, because the specimens used for this assessment were drawn from the same experimental program that informed the development of the model, the present comparison does not constitute a fully independent validation. Further verification against independent experimental datasets covering broader material, geometric, and loading parameters is therefore warranted.

### 4.6. Practical Considerations and Future Research

The practical implementation of the proposed HSSWM–ECC strengthening system requires coordinated control of substrate preparation, strand anchorage, mesh installation, ECC placement, and curing. Effective interfacial force transfer depends strongly on the condition of the existing concrete substrate; deteriorated or loose concrete should therefore be removed, followed by adequate surface roughening and cleaning before application of the ECC layer. Construction around beam–column intersections and member corners may be constrained by limited accessibility, potentially affecting surface treatment, anchorage installation, and continuity of the steel-strand mesh. Quality control should therefore include verification of substrate soundness and cleanliness, anchorage integrity, strand alignment and spacing, strengthening-layer thickness, adequate ECC placement and consolidation, and compliance with the prescribed curing regime. In the present tests, no premature anchorage failure or extensive interfacial debonding was observed before peak resistance, although localized ECC bulging developed near the beam–column interface at advanced loading stages, indicating the sensitivity of this region to local interface quality and construction consistency. Because the present investigation was conducted under controlled laboratory conditions, the long-term performance of the ECC–concrete interface, steel strands, and anchorage system under sustained environmental exposure remains to be established.

Beyond ambient service conditions, the response of HSSWM–ECC-strengthened joints under elevated temperatures and fire exposure represents an important extension of the present work. Fire-induced thermal gradients and temperature-dependent degradation of concrete, ECC, steel reinforcement, and steel strands may modify interfacial stress transfer and the composite action developed within the strengthened section. Previous studies on RC members exposed to standardized fire have demonstrated the importance of transient temperature distributions and temperature-dependent material properties in evaluating structural response [32]. Future investigations should therefore address the coupled thermal–mechanical behavior, interface integrity, anchorage performance, and post-fire residual flexural capacity of HSSWM–ECC-strengthened beam–column joints.

## 5. Conclusions

This study experimentally investigated eight RC beam–column joint specimens to evaluate the effects of pre-damage level, steel strand mesh spacing, and axial compression ratio on the beam-end flexural behavior of HSSWM-ECC-strengthened joints. Based on the experimentally identified load-resisting mechanisms, an analytical method was further developed to predict the beam-end flexural capacity. The main conclusions are as follows:

(1) All strengthened specimens were governed by beam-end flexural failure, while the columns and joint cores remained essentially intact. The HSSWM-ECC strengthening layer developed fine and distributed cracks at the beam ends, with no extensive debonding or anchorage failure observed before the peak load. The fiber-bridging action of ECC and the continuous tensile resistance provided by the steel strand mesh jointly suppressed dominant-crack localization and concrete spalling. Moreover, the strengthening layer continued to provide additional tensile resistance after yielding of the beam longitudinal reinforcement, thereby delaying post-peak strength degradation.

(2) HSSWM-ECC strengthening increased the peak loads of the specimens by 39.24–63.88% compared with the unstrengthened specimen. Reducing the steel strand spacing from 70 to 30 mm increased the peak load by 8.86%, while the corresponding capacity enhancement relative to the unstrengthened specimen increased from 50.55% to 63.88%. This indicates that increasing the effective tensile area of steel strands per unit width can substantially enhance the flexural contribution of the strengthening layer.

(3) Pre-damage level and axial compression ratio affected the strengthened response in different ways. A higher pre-damage level resulted in a more pronounced reduction in the subsequent flexural response of the strengthened joint. The peak load of the lightly pre-damaged specimen was only 1.44% lower than that of the undamaged strengthened specimen, whereas a reduction of 11.28% was observed at the higher pre-damage level. For axial compression ratios of 0.2–0.4, the differences in peak load remained within 3.64%, indicating a relatively limited influence on peak flexural resistance within the investigated range. In contrast, increasing the axial compression ratio within this range was associated with more rapid post-peak strength degradation and a reduction in ultimate deformation capacity.

(4) Based on the plane-section assumption and sectional force equilibrium, an analytical method was developed to predict the beam-end flexural capacity of pre-damaged RC beam–column joints strengthened with HSSWM-ECC. The effects of the pre-damage history were represented by a stiffness reduction coefficient and a stress reduction coefficient, while a side-layer utilization coefficient was introduced to account for the additional contribution of the wrap-around strengthening layer. Neglecting the contribution of the side strengthening layers reduced the calculated capacity by 1.8–7.1%. Assessment against the seven strengthened specimens showed that the deviations between the calculated and experimental capacities were within 7%, indicating that the proposed model can reasonably capture the beam-end flexural capacity within the investigated parameter range.

The findings and the proposed analytical method are primarily applicable to HSSWM–ECC-strengthened RC beam–column joints governed by beam-end flexural failure under monotonic loading, within the investigated conditions comprising undamaged and pre-damaged states with prescribed drift ratios up to 1.50%, steel strand spacings of 30–70 mm, and axial compression ratios of 0.2–0.4. Extension to joint-panel shear failure, substantially different strengthening configurations, broader parameter ranges, or reversed cyclic loading requires further experimental verification.

## Figures and Tables

**Table 1:** Design parameters of specimens.

Specimen ID	Pre-Damage Drift Ratio (Target Displacement)	Steel Strand Spacing/mm	Axial Compression Ratio
S-0-0-0.3	-	-	0.3
S-0-50-0.3	-	50	0.3
S-0-30-0.3	-	30	0.3
S-0-70-0.3	-	70	0.3
S-16-50-0.3	0.75% (16 mm)	50	0.3
S-32-50-0.3	1.5% (32 mm)	50	0.3
S-0-50-0.2	-	50	0.2
S-0-50-0.4	-	50	0.4

Note: The pre-damage levels were defined by the prescribed column-top drift ratios during monotonic pre-loading. Drift ratios of 0.75% and 1.50% correspond to target horizontal displacements of 16 mm and 32 mm, respectively. These values characterize the imposed pre-loading histories rather than the residual damage states after unloading.

**Table 2:** Mechanical properties of materials.

Materials		Compressive Strength (MPa)	Yielding Strength (MPa)	Tensile Strength (MPa)	Ultimate Tensile Strain (%)	Elastic Modulus (×10^5^ MPa)
Concrete		33.2	/	/	/	0.31
Steel bars	Φ8	/	328.7	540.2	/	2.11
	Φ14	/	419.3	625.4	/	2.13
	Φ16	/	455.6	637.1	/	2.16
Steel strand		/	/	1557	2.89	1.43
ECC		48.87	/	4.60	3.85	/

Note: Φ denotes the reinforcing bar diameter.

**Table 3:** Mix proportions of ECC.

Cement	Sand	FA	SF	Water	PVA	WR	HPMC
1	0.4	3	0.073	1.02	0.072	0.0407	0.0018

**Table 4:** Results of the lateral-side influence of the strengthening layer on bearing capacity.

Specimen ID	Theoretical Calculation Value (kN)	Ignore the Lateral-Side Influence of the Strengthening Layer (kN)	Reduction (%)
S-0-50-0.3	56.53	52.95	6.30
S-0-30-0.3	60.29	58.78	2.50
S-0-70-0.3	52.01	48.58	6.60
S-16-50-0.3	55.41	53.01	4.30
S-32-50-0.3	51.53	50.61	1.80
S-0-50-0.2	56.96	52.95	7.10
S-0-50-0.4	56.91	52.95	7.00

**Table 5:** Bearing capacity results from theoretical calculations.

Specimen ID	*P_t_* (kN)	*P_c_* (kN)	Theoretical-to-Test Ratio
S-0-50-0.3	54.71	56.53	0.968
S-0-30-0.3	57.13	60.29	0.948
S-0-70-0.3	52.48	49.59	0.945
S-16-50-0.3	53.92	55.41	0.973
S-32-50-0.3	48.54	51.53	0.942
S-0-50-0.2	53.35	56.96	0.937
S-0-50-0.4	55.29	56.91	0.972

## Data Availability

The original contributions presented in this study are included in the article. Further inquiries can be directed to the corresponding author.
